# Objective risk assessment vs standard care for acute coronary syndromes—The Australian GRACE Risk tool Implementation Study (AGRIS): a process evaluation

**DOI:** 10.1186/s12913-022-07750-8

**Published:** 2022-03-22

**Authors:** Janice Gullick, John Wu, Derek Chew, Chris Gale, Andrew T. Yan, Shaun G. Goodman, Donna Waters, Karice Hyun, David Brieger

**Affiliations:** 1 Susan Wakil School of Nursing & Midwifery, Faculty of Medicine and Health, University of Sydney, Sydney, NSW Australia; 2 Susan Wakil School of Nursing & Midwifery, and Site Services, University of Sydney Library, University of Sydney, Sydney, NSW Australia; 3 College of Medicine and Public Health, Flinders University of South Australia, Adelaide, Australia; 4 Leeds Institute of Cardiovascular and Metabolic Medicine, University of Leeds, Leeds, England; 5 Department of Medicine, University of Toronto, St Michael’s Hospital, Toronto, ON Canada; 6 Canadian VIGOUR Centre, Department of Medicine, University of Alberta, Edmonton, Canada; 7 School of Health Sciences, Faculty of Medicine and Health, University of Sydney, Sydney, Australia; 8 Concord Repatriation General Hospital, ANZAC Research Institute, Concord West, Australia; 9 Concord Clinical School, Concord Repatriation General Hospital, ANZAC Research Institute, Concord West, Australia; 10 Faculty of Medicine and Health, University of Sydney, Sydney, +61 2 9767 5000 Australia

**Keywords:** Acute coronary syndromes, GRACE Risk Tool, Implementation, Implementation fidelity, Process evaluation, Risk stratification, Quality of care, Theoretical Domains Framework, COM-B, Behaviour Change Wheel

## Abstract

**Background:**

Structured risk-stratification to guide clinician assessment and engagement with evidence-based therapies may reduce care variance and improve patient outcomes for Acute Coronary Syndrome (ACS). The Australian Grace Risk score Intervention Study (AGRIS) explored the impact of the GRACE Risk Tool for stratification of ischaemic and bleeding risk in ACS. While hospitals in the active arm had a higher overall rate of invasive ACS management, there was neutral impact on important secondary prevention prescriptions/referrals, hospital performance measures, myocardial infarction and 12-month mortality leading to early trial cessation. Given the Grace Risk Tool is under investigation internationally, this process evaluation study provides important insights into the possible contribution of implementation fidelity on the AGRIS study findings.

**Methods:**

Using maximum variation sampling, five hospitals were selected from the 12 centres enrolled in the active arm of AGRIS. From these facilities, 16 local implementation stakeholders (Cardiology advanced practice nurses, junior and senior doctors, study coordinators) consented to a semi-structured interview guided by the Theoretical Domains Framework. Directed Content Analysis of qualitative data was structured using the Capability/Opportunity/Motivation-Behaviour (COM-B) model.

**Results:**

*Physical capability* was enhanced by tool *usability*. While local stakeholders supported *educating frontline clinicians,* non-cardiology clinicians struggled with specialist terminology. *Physical opportunity* was enhanced by the paper-based format but was hampered when busy clinicians viewed risk-stratification as *one more thing to do*, or when form *visibility* was neglected. *Social opportunity* was supported by a *culture of research/evidence* yet challenged by *clinical workflow* and *rotating medical officers*. *Automatic motivation* was strengthened by *positive reinforcement*. *Reflective motivation* revealed the GRACE Risk Tool as *supporting* but potentially *overriding* clinical judgment. *Divergent professional roles and identity* were a major barrier to integration of risk-stratification into routine Emergency Department practice. The cumulative result revealed *poor form completion behaviors* and a failure to embed risk-stratification into routine patient assessment, communication, documentation, and *clinical practice behaviors*.

**Conclusions:**

Numerous factors negatively influenced AGRIS implementation fidelity. Given the prominence of risk assessment recommendations in United States, European and Australian guidelines, strategies that strengthen collaboration with Emergency Departments and integrate automated processes for risk-stratification may improve future translation internationally.

## Background

Acute coronary syndromes (ACS) are a major cause of morbidity, hospital admission, and mortality placing considerable burden on health systems. Evidence-based therapies informed by timely, structured risk-stratification may improve patient outcomes. Paradoxically, however, lower-risk patients more often receive guideline-recommended, in-hospital care including early invasive strategies and more aggressive drug therapy [1]. This inconsistency is attributed to subjective risk-assessment that underestimates coronary risk and over-estimates risk from cardiac procedures [1–3].

As care variance is associated with higher cardiac event and death rate [1, 3], United States, European and Australian clinical guidelines recommend routine use of validated ischaemic/bleeding risk-assessment instruments to assist decisions about invasive strategies for ACS [4–6]. Given the weak level of evidence [7] for this recommendation (IIIB), the Australian GRACE Risk score Intervention Study (AGRIS) aimed to test the evidence for routine risk-stratification using the GRACE Risk Tool in patients admitted with ACS [8].

AGRIS was a prospective cluster [hospital-level] randomised, open-label, blinded, endpoint (PROBE) trial of 24 Australian hospitals comparing use of an evidence-based ischaemic/bleeding risk-stratification tool to usual care. The AGRIS rationale and design is published elsewhere [8]. Briefly, the key aims were to: 1) reduce mismatch between individual patient risk, ACS management and clinical outcomes by embedding ischaemic/bleeding risk assessment using the GRACE Risk Tool (GRT) and evidence-based treatment recommendations into routine clinical practice, and 2) comparing patient outcomes between active (*n* = 12) and control (*n* = 12) arms. Outcomes of interest were hospital performance (receipt of early invasive strategies, prescription of 4 of 5 guideline-recommended medications, cardiac rehabilitation referral) and all-cause mortality, myocardial infarction (MI), and a composite of mortality or MI at 12-months [8].

The GRT which underpins this practice change combines two validated risk scores to guide ACS treatment decisions [8]: the GRACE Risk Score (GRS) is a compilation of clinical risk-stratification measures (age, heart rate, systolic blood pressure, creatinine, Killip class, ST-segment deviation on ECG, troponin and cardiac arrest event) to assess risk for in-hospital and 6-month mortality [9]; the CRUSADE score uses a compilation of factors to assess major bleeding risk in patients presenting with Non-ST segment Elevation Myocardial Infarction (NSTEMI), before commencement of treatment (heart rate, systolic blood pressure, haematocrit, creatinine clearance, sex, clinical signs of congestive heart failure, peripheral vascular disease and diabetes mellitus) [10].

The analysis of AGRIS ran parallel to, and independent of, this implementation process evaluation. The AGRIS trial found that among 2,318 patient participants, 63% were at high ischaemic risk. The overall rate of invasive management for ACS was higher among hospitals in the active arm, 92% versus 84% (adjusted odds ratio 2.26 [95% C.I.: 1.30–3.96], p = 0.004), and invasive therapy was higher for younger patients at lower ischaemic risk. However, there was no overall difference in: prescription of 4/5 guideline-recommended medications; cardiac rehabilitation referral; provision of all three hospital performance measures for high ischaemic-risk patients; MI; nor, mortality at 12-months [11]. After 4.5 years, interim analysis led to early cessation of AGRIS based on futility to identify a difference between groups [12].

There are several possible contributors to this result. Larger sample sizes are often necessary to address variability in complex interventions [13]: while no difference in secondary prevention, hospital performance or clinical outcomes were observed, this was in the context of a trial stopped before recruitment completion, due to futility. Across groups, clinical performance was better than expected and event rates were low; the hospitals recruited for AGRIS demonstrated higher rates of guideline-indicated therapies than was anticipated, based on historical data from the CONCORDANCE Registry. These factors may have reduced the power to detect meaningful differences [14]. A limitation of the original trial may be the application of the GRT to STEMI patients (one-third of participants), given that in STEMI, early angiography is not influenced by risk-stratification tools.

A further explanation for neutral results may be the quality of implementation fidelity; the degree to which an intervention is delivered as intended [15]. Poor implementation fidelity can shape the relationship between the intervention and its outcomes, leading to erroneous conclusions about the intervention’s effectiveness [13, 16]. This paper aims to understand implementation fidelity (the barriers and enablers to implementation of the GRT into routine clinical workflow) and in doing so, to allow a more nuanced reflection on AGRIS findings [14].

## Methods

### Design

**Fig. 1:**
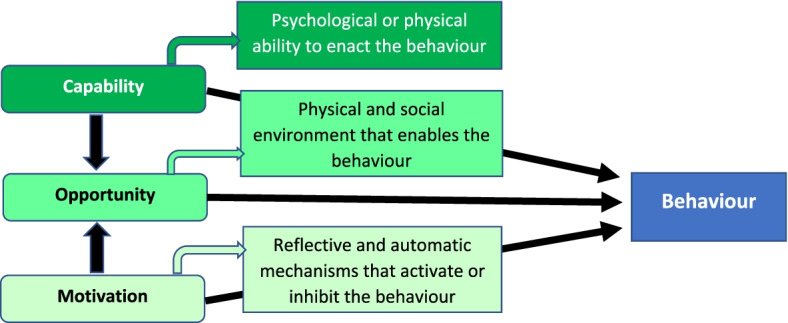
The COM-B model (reproduced with permission [20])

This multi-centre, cross-sectional, descriptive qualitative study used semi-structured interview data. Directed Content Analysis [17] guided by Michie et al. [18] Capability/Opportunity/ Motivation-Behaviour (COM-B) model structured qualitative analysis (Fig. 1). The COnsdolidated criteria for REporting Qualitative research (COREQ) [19] guided reporting rigour.

### The AGRIS implementation strategy

While strategies for implementation were expected to vary between hospitals according to local structure, resources and needs, two important implementation principles were pivotal: 1) Adjustment of ACS admission process to include GRT risk-stratification 2) Communication of the clinical utility and relevance of the GRT and treatment recommendations to the entire multi-disciplinary team, specific to their roles.

Key tasks were that staff calculate both the GRS and CRUSADE Score to estimate risk of recurrent MI or death, and bleeding respectively; classify the patient as low, intermediate or high-risk; use this classification to communicate treatment recommendations using the GRT nomogram; and documentation of subsequent recommended treatment as either “indicated”, “not indicated, or “contraindicated” by admitting medical or nursing staff [8]. Stages of implementation appear in Table 1.

**Table 1:** Stages of implementation

Implementation stage	Activities
Initial AGRIS Study information	For the substantive AGRIS study, clinicians representing the 43 sites enrolled in the Cooperative National Registry of Acute Coronary care, Guideline Adherence, and Clinical Events (CONCORDANCE registry) were briefed on the study protocol during an annual CONCORDANCE Investigator meeting
Inclusion criteria for AGRIS	Twenty-four CONCORDANCE sites were invited to participate based on: 1) an Emergency Department with 24/7 access, 2) without an existing, embedded ACS risk-stratification/decision-support tool, and 3) cardiology/medical units willing to implement the GRACE Risk Tool and associated treatment plan into the routine care processes. Further considerations were adequate perceived clinical leadership, perceived openness to change, existing measurement for system and process evaluation, and networks for support and referral between rural, regional and metropolitan hospitals [8]
Implementation team development	Strategic teams and role descriptions were developed, internal and external to the CONCORDANCE team and participating sites [8]. The CONCORDANCE staff were not to be involved in implementing the GRACE Risk Tool; their role was restricted to data collection
Implementation planning	In the pre-implementation phase, initial meetings between the study implementation steering committee and senior hospital staff (the implementation team) identified and engaged all affected multidisciplinary staff within the emergency and cardiology departments of all hospitals (n = 12) randomised to the active arm of AGRIS to discuss strategies to facilitate implementation
Implementation phase	In the implementation phase, the GRACE Risk Tool and associated treatment plan was introduced into hospital workflow at these 12 hospitals. All 12 hospitals reached the threshold for inclusion as an active site (defined as 90% completion of the GRACE Risk Tool in consecutive patients also enrolled in the CONCORDANCE registry in any one month: the CONCORDANCE registry seeks to recruit the first ten consecutive ACS patients per month presenting to participating facilities) [8]. The CONCORDANCE Registry functioned as the data spine for AGRIS. The intention was that all patients presenting with ACS would be assessed using the GRACE Risk Tool, and uptake would be confirmed by the presence of that completed worksheet in the subset enrolled in CONCORDANCE. This smaller CONCORDANCE cohort would be the group analysed for outcomes. Data collection was to be undertaken by CONCORDANCE study coordinators who were to be independent of clinicians and others involved in the intervention roll-out [8]
Post-implementation	Further ethical approval was obtained for the process evaluation (CH62/6/2013–154). Key stakeholders from five of the 12 hospitals to engage in an in-depth interview to provide feedback on implementation processes at their site, and to consider barriers and enablers to effective and sustained implementation of the intervention into wider cardiology practice

### Sample and setting

The process evaluation sought participation from five hospitals from the active arm of AGRIS. Seven hospitals from three Australian states were invited using purposive sampling seeking maximum variation [21] for geographical setting (city versus rural/regional), level and complexity of clinical services, and a diversity of implementation success based on the number of GRT forms completed (including the least and most successful available sites)(Table 2). Two sites in the lowest range of success declined participation: one site did not provide a reason, and a second declined, citing the burden of local submission of a revised ethics protocol.

**Table 2:** Characteristics of the hospitals participating in AGRIS process evaluation

Participating Site	Site characteristics
Hospital 1	Publicly funded, tertiary urban teaching hospital in a large Australian city. 800–1000 beds. 24-h PCI-capable cardiac catheter laboratory, Coronary Care Unit, ED-based Cardiac Investigation Unit
Hospital 2	Publicly funded, regional teaching hospital, + 150 beds, Critical care unit with dedicated coronary care beds. Cardiac catheter laboratory offering diagnostic procedures only. Distance from closest state capital city > 150 km
Hospital 3	Publicly funded, regional teaching hospital, 500 + beds with coronary care unit. 24-h PCI-capable cardiac catheter laboratory, Distance from closest state capital city > 75 km
Hospital 4	Tertiary, publicly funded tertiary teaching hospital in a large Australian city. 800–1000 beds. 24-h PCI-capable cardiac catheter laboratory, Coronary Care Unit, ED-based Cardiac Investigation Unit
Hospital 5	Publicly funded, regional teaching hospital. Critical care unit with dedicated coronary care beds. 24-h PCI-capable cardiac catheter laboratory, Distance from closest state capital city > 75 km

#### Participant characteristics

Participants (*n* = 16) were clinicians and local study coordinators considered key informants in the local implementation of AGRIS. Key stakeholders across active sites were aware, prior to implementation, of the AGRIS process evaluation phase and of possible contact for interview. Once the five participating sites were confirmed, local study coordinators were invited to participate and emailed a participant information and consent form. They then contributed to snowball sampling by inviting other local stakeholders who were forwarded recruitment documentation and invited to contact a researcher (JG) to discuss participation.

Participants included cardiologists (*n* = 3) (including two heads of department); cardiology registrars (basic physician trainees [*n* = 2], advanced cardiology trainees [*n* = 2]); advanced practice cardiology nurses [22] (*n* = 3); and study coordinators (*n* = 5) who had clinical backgrounds or concurrent practice in cardiology (predominantly nursing) (Table 3).

**Table 3:** Role descriptors of participants

Position description	Definitions as applied to participants in this study
Head of Department^(HoD)^	A cardiologist who is the medical director of a cardiology department
Cardiologist^(Card)^	An interventional cardiologist employed as either a hospital staff specialist or visiting medical officer
Advanced Trainee^(AT)^	A medical officer employed in a role that is the culmination of the minimum 6-year training program preparing them to become a physician and Fellow of the Royal Australian College of Physicians within a specialist area of medical practice; in this case, Cardiology
Basic Physician Trainee ^(BPT)^	A medical officer employed in a role that prepares them for Part 1 of the Royal Australian College of Physicians examination
Intern^(Int)^	A medical officer undertaking a period of mandatory, supervised general clinical practice, allowing graduates to apply and consolidate their clinical skills
Advanced Practice Nurse^(APN)^	Advanced Practice Nurses are employed in a range of roles – in this study, APNs were working as Clinical Nurse Consultants (incorporating direct clinical consultancy, education, research, clinical leadership and support of systems), or Cardiac liaison roles between Emergency and Cardiology Departments to facilitate rapid assessment, and either departmental transfer or discharge
Clinical Trial Coordinator^(CT Coord)^	Study coordinators who worked within their institutions to support the protocol of the AGRIS trial. In this study, all coordinators had previously worked in clinical roles in acute clinical settings

### Data collection

An interview guide was constructed using the Theoretical Domains Framework; a validated list of expert consensus-derived theoretical constructs known to influence behaviour change [23]. This framework easily intersects with the Behaviour Change Wheel, enabling reflection upon *Capability*, *Opportunity*, and *Motivation* alongside policy categories and implementation functions to design/explain new *Behaviours* (Table 4) [18, 23]. Once implementation was considered well-established, interviews were conducted by a nurse academic (JG) with experience as an advanced practice cardiology nurse and expertise in qualitative research, including ACS systems of care. No author involved in participant recruitment, nor interviewing, had a pre-existing relationship with any participant. Participants provided a single, semi-structured interview either face-to-face (*n* = 4), or by phone (*n* = 12) in a private space in the clinical setting. Median interview duration was 26-min. Interviews were audio-recorded and transcribed verbatim with supporting field notes taken immediately after interview. Analysts were blinded to AGRIS trial results.

**Table 4:** Mapping the Behaviour Change Wheel’s COM-B system to the Theoretical Domains Framework (reproduced with permission [24])

**Com-B Component (for analysis)**		**TDF Domains (to structure interview guide)**
**CAPABILITY**	Physical	Skills
	Psychological	Knowledge Skills Memory, attention and decision processes Behavioural regulation
**OPPORTUNITY**	Social	Social Influences
	Physical	Environmental context & resources
**MOTIVATION**	Reflective	Social/professional role & identity Beliefs about capabilities Optimism Beliefs about consequences Intentions Goals
	Automatic	Social/Professional role & identity Optimism Reinforcement Emotion

### Data analysis

We drew on the methods of Directed Content Analysis [17], guided by the COM-B model [18] to analyse verbatim interview data. The COM-B model is a theoretical construct, developed through synthesis of 19 frameworks of behaviour change identified through systematic literature review. Inter-rater reliability (79–88%) and concurrent validity (75–85%) of the COM-B as a coding structure was confirmed by the framework developers [18], and the model has since been recommended for use by government agencies [25] and the World Health Organisation [26], with published application to a broad range of clinical contexts [27–30].

Operational definitions were established for the theoretical categories of the COM-B (Fig. 1): *Capability* (physical/psychological) is the knowledge and skills that enable people to enact a behaviour; *Opportunity* (physical/social) refers to external mechanisms that activate or inhibit a behaviour; and *Motivation* (automatic/reflective), influenced by both capability and opportunity, energises and drives behaviours based on habit or contemplative reasoning (e.g., values/beliefs). Subsequent *Behaviours* were then explored as outcomes arising from the COM components. Data were coded using these predetermined categories by two doctorally-prepared researchers not otherwise involved in AGRIS (JG, JW). Within these categories, data were further themed inductively to produce a detailed description of the factors that enabled or obstructed implementation of AGRIS for risk-stratification, documentation and subsequent clinical reasoning and action. The resulting description was ‘rich’ with a well-rounded explanation resulting from diverse cases within key themes, and ‘thick’, with multiple exemplars available for theme construction and confirmation [31].

**Fig. 2:**
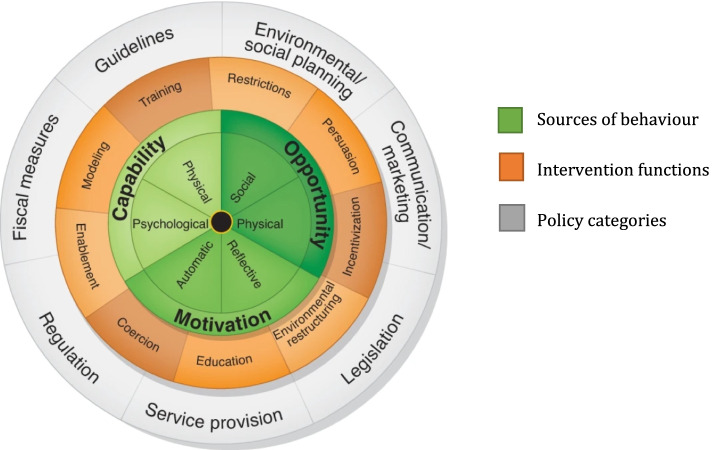
The Behaviour Change Wheel (Reproduced with Permission [32])

Finally, we discuss the implications of our findings with reference to the Behaviour Change Wheel [18]. Designed to intersect with the COM-B, the Behaviour Change Wheel proposes a range of possible *intervention functions* (orange band, Fig. 2) and *policy categories* (grey band, Fig. 2) providing structure for reflection on local implementation.

## Results

In the reporting of findings, participants are indicated by pseudonym, followed by the superscript number of their employing hospital (Table 2), and their role descriptor (Table 3).

### Physical Capability

*Physical capability* refers to the skills required to complete the target behaviour, specifically, completion of the GRT [18].

#### GRT usability

Most participants raised the ease of the GRT format. Thea^1(BPT)^ noted this simplicity: *“It's all on one sheet… a few numbers, you circle and add them… anyone could do it.”* Beth^2(APN)^ noted the form format presented occasional difficulty integrating two risk scores on the one document: *“Staff didn't know… if they had a high GRACE score, but… an intermediate CRUSADE score, which colour they should* [prioritise]*”.*

### Psychological Capability

*Psychological capability* refers to the knowledge and thought processes required to make application of the new skill possible [18].

#### Clinician capability

Regardless of their role in the health system and AGRIS, all participants confirmed their own *psychological capability,* explaining in detail, the purpose and potential clinical utility of AGRIS. Jared^2(Card)^ believed AGRIS was most useful for junior or non-cardiology doctors: *“Cardiologists and trainees do [risk-stratification] automatically. But general medical rotators… don’t see the wood from the trees… if you give them a format… it’s in front of their face.”* However, other non-cardiology clinicians had difficulty calculating and documenting the GRT scores: Beth^2(APN)^ explained: *"It baulks people… because we're not an interventional hospital. It talks about… primary PCI… medications we don't use… If you're not familiar with specialty terminology, it puts up barriers.”*

#### Education of frontline clinicians

The solid education and support provided by the central AGRIS team were noted across participating hospitals, while information sessions led by influential cardiologists lent credibility. These introductory sessions were then followed by connecting central AGRIS coordinators with local study coordinators and implementation leads to ensure familiarity with study processes. After initial facility training, local study personnel arranged education for rotating medical clinicians with varying levels of engagement. Fran^5(CT Coord)^ explained: *“We had all the evidence available… as we trained JMOs… ‘Here's the background… the paperwork’. Some looked at it. Most didn't, but some said, ‘Okay, now I'd like to know this’…”.*

Medical trainees Seb^3(AT)^ and Chloe^5(Int)^ felt they missed some initial and ongoing training: *“…it's probably issue of our availability… our busyness and not being around.”* Sam^1(HoD)^ provided a caveat to the impact of education: *“I think we've educated our trainees… The difficult thing is getting the patients’ GRACE score into the conversation and into routine practice.”*

Because some frontline clinicians’ had competing demands and priorities, local champions ‘selling the message’ became an important part of *psychological capability*. Abigail^2(CT Coord/APN)^ explained: *“I did a bit of reading… so I could throw some numbers at people… When you say ‘Well, it’s reducing mortality and we’re all here for our patients…’, some people are like, ‘Oh, yeah, I see that. Show me again’.”*

### Physical opportunity

*Physical opportunity* requires access to the necessary materials, and time to enact the target behaviour [18].

#### One more thing to do

Across institutions, more than half the participants described a wide perception of risk-stratification as “one more thing to do”. This was particularly, but not exclusively the case when engaging Emergency Department (ED) clinicians. Beth^2(APN)^ explained: *"ED is at saturation point… ‘It's just another form’. They see it as more work and they're not coping with what they’ve got… they’re drowning*”. This was confirmed by ED physicians. Jerome^2(AT)^ explained: *“When I asked some ED doctors why they weren't doing it routinely the most common response was that they didn't have time”.*

#### GRT visibility

*Physical opportunity* depended on the GRT’s visibility and availability: it was much more likely to be incorporated into daily workflow when it was ready-to-hand. Jerome^2(AT)^ explained how the lack of form visibility was a barrier: *“…it would get lost easily… When I went to ED to find the AGRIS scoring sheet… they were under a mannequin”.* Fran^5(CT Coord)^ described how the set-up of AGRIS as a research study hampered GRT visibility and access, with lack of clarity about whether to file the GRT with the progress notes or research files: *“We were instructed… to keep it with the patient files, but it became hard… duplicating things, because [research coordinators] have to have copies.”*

#### Paper versus electronic format

Participating hospitals had varying degrees of EMR establishment and none used online ACS order sets. The growing prominence of the electronic medical record (eMR) stimulated discussion about the paper-based GRT format and its impact on *physical opportunity*. Maree^5(APN)^ appreciated the tangible nature of the paper-based format for fostering clinician engagement: *“We’d say, "We'll put [the GRT] in a folder for you… Go through these and use them for your decision-making." Whereas, if they're electronic, you need to go into each patient record to see that.*” Jared,^2(Card)^ saw a future in automated curation of GRT data in the eMR to enhance *physical capability*: *“Most of the… score should be self-populating… any decent computer programmer* [should enable automatic calculation] *so it hits you in the face”.*

### Social Opportunity

*Social opportunity* refers to how conducive the socio-cultural milieu is to the target behaviour [18].

#### A culture of research and evidence

The high initial acceptance of AGRIS in Cardiology was attributed to a strong research culture. Meredith^3(BPT)^ reflected: “*Consultants would like us to contribute [to research]… that’s the biggest enabler*.”

#### Clinical workflow

Sometimes, completion of the GRT required information that was not available because of clinical workflow factors. Thea,^1(BPT)^ revealed: *“…country patients arrive who we don't yet have details [for]… So we have to chase their presentation heart rate, blood pressure… so [the GRT] can't be done on the fly in the Cath Lab”.*

Sandra^3(APN)^ explained further: *“It’s workflow… the way the doctors round is a pretty big barrier, it’s rushed… they're thinking of a hundred things, so the [GRT] falls way behind in priority”.* This was compounded when complex patients were managed in non-Cardiology areas meaning some risk assessment was missed. A further workflow impact was explained by Oliver^(HoD)^: in his institution, it was the seasonal variation in ACS presentations that dictated angiogram capacity, reducing the relevance of the GRT during the winter peak.

#### Rotating medical officers

Despite ongoing systematic training, the routine rotation of junior medical officers challenged *social opportunity* due to a loss of project momentum. Jerome^2(AT)^ explained: *“Initially, it had an uptake of about 75%… as the first set of BPTs were leaving, it dropped to about 30%. Second set of BPTs, maximum 20%.”* Across participating hospitals, addressing this decline was difficult a when new medical staff already felt overwhelmed by new information.

### Automatic motivation

*Automatic motivation* refers to the elements that enable the target behaviour to become habitual [18].

#### Reinforcement as an enabler

*Automatic motivation* to complete the GRT required constant reinforcement through incentives and reminders, and this was frequently led by local study coordinators. Fran^5(CT Coord)^ used incentives with new staff:

*…developing relationships as [medical officers] changed…. we'd have coffee vouchers... a nice spread of food… For them to be able to… sit, have a coffee… listen to what we had to say, then go to their next job… lowered [our impact on] their workload.*

Reinforcement from senior medical staff was also valued. Jerome^2(AT)^ explained: *“If I came across someone in the hallway I’d say, ‘Just remember to use the AGRIS criteria’.”* One facility had introduced an innovative reinforcement system where the GRACE/CRUSADE scores were visible on an eMR inpatient list.

### Reflective motivation

*Reflective motivation* is dependent upon people’s intentions to perform, and positive or negative beliefs about the value of the target behaviour [18].

#### Support for clinical judgement

For regional hospitals, *reflective motivation* was strengthened by the GRT’s capacity to support clinical judgement, informing triage and transfer decisions. Beth^2(APN)^ explained: *“…it's very useful to figure, ‘Hang on, this patient is higher-risk than we thought… let's get them to an interventional hospital sooner.”*

Fran^5(CT Coord)^ raised the value of the GRT in discharge-planning: “*From a medico-legal perspective, we want to be sure. A lot of patients, we're sending back home… 600-800 km from here”.* Jared^2(Card)^ expanded: *“It’s not just calculating the score, it’s when… it says, ‘According to this score, you should be doing this’.”* Even in tertiary centres, doctors were surprised at the level of some patients’ risk, while others found it captured best practice by prompting a rationale when recommended treatment was omitted.

Equally, some senior physicians were concerned that the GRT could override good clinical judgement. Jerome^2(AT)^ revealed: *“…if someone's 85-years-old… Their co-morbidities might not be covered by the GRT… It’s a tool. It shouldn't take the place of a clinician’s decision”.* Sam^1(HoD)^ believed the GRT merely replicated good clinical judgement: *“If people practice good clinical medicine, this study shouldn't make any difference.”*

#### Divergent professional roles and identity

There were differing approaches to who completed the GRT according to *professional roles and identity:* (ED *versus* Cardiology clinicians; and medical *versus* nursing staff *versus* study coordinators). The AGRIS investigators intended that ideally, an admitting doctor would complete the form, calculate the risk score and use it to inform and communicate clinical judgement. Jared^2(Card)^ believed that ED involvement was vital to the success of AGRIS: *“In our situation [regional hospital]… We ‘drip and ship’: thrombolyse and transfer, so you need ED to be involved.”* While some facilities planned for ED to be the primary site for form completion, this was universally unsuccessful. Beth^2(APN)^ explained*: “It was to be started in ED but… We got a lot of resistance, so… cardiology teams do it because we've got 24-h to get the form done”.*

Difficulty engaging leadership from senior ED clinicians impeded *reflective motivation*. Abigail^2(CT Coord/APN)^ recalled: *“The Director of Emergency [is] like, ‘Nah, not doing it’. It's really hard then. Everyone's like, ‘Well if he thinks it's… not important’, then it's just some nurse saying it”.* Participants from three facilities suggested the divergence between departmental approaches arose from a systematic lack of structured risk-documentation in ED, and all raised the Chest Pain Pathway as a similar tool that remained unembedded. Sandra^3(APN)^ explained: *“Our [ED] Chest Pain Pathway compliance rate is 2% or something ridiculous. So if that isn't being done…".*

The differing evidence bases between ED and Cardiology also seemed to underpin clinicians’ *professional identity* and feed into their motivation and intention to harmonise clinical practices. Jared^2(Card)^ explained: *“If you look at [evidence] within cardiology and ED literature, they’re often diametrically opposed. It’s bizarre. There’s all sorts of things we wouldn’t agree with.”* Two participants felt the GRT’s clinical fit for ED was not optimal. Maree^5(APN)^ explained: *“[GRT] is not as easily applied in an emergency setting… they would prefer a HEART or EDACS score. They tend to follow their own cohort of experts… So, GRACE is fairly distinctly for cardiology users”*.

Due to resistance from ED clinicians across sites, Cardiology teams took primary ownership of GRT completion. Role modelling and leadership from Heads of Department were key in promoting *reflective motivation*. Sam^1(HoD)^ explained his clear expectations as a leader, and the resulting compliance and impact on clinical reasoning and autonomy. *“The registrars have to do it … because I told them to do it… and I think that gave them more motivation to think ‘I should push for this patient having an angiogram’.”* This leadership was appreciated at all levels and, along with adequate human resources, seemed to explain why some departments found implementation easier. However, leadership needed to be sustained and engaged. Beth^2(APN)^ explained: *“[HoDS] assumed their doctors were doing it, but it's not something they policed.”* At one hospital, the head of the Cardiology department was unaware that implementation had begun.

In most hospitals, effective leadership also came from advanced practice cardiology nurses (APNs) who brought skills in education and systems improvement. Despite the research protocol stipulating that implementation be led by clinical staff, study coordinators also completed the forms or recruited other nursing staff to do so to increase completion rates. In fact, most facilities took a pragmatic approach and eventually accepted any reasonable person to complete the GRT to meet the minimum number required per-month (nine) under the AGRIS protocol. One limitation of APN and research coordinator leadership was that there were fewer APN roles in regional hospitals, and many APNs and study coordinators worked part-time, impacting the 24-h completion goal. This led to retrospective form completion, with obvious issues for the GRT’s wider application in early clinical risk-stratification and care-planning.

### Resulting behaviours

#### Form completion behaviours

All facilities reported difficulty maintaining *form completion behaviours*. This was corroborated by a mean GRT non-completion rate of 33% across the five process evaluation sites, and a mean non-completion rate of 41% across the 12 active arm sites (for the subset of patients also enrolled in the CONCORDANCE Registry). The frequency of non-completion was distributed evenly across tertiary, metropolitan and regional hospitals. One reason for the lack of embedded scoring behaviours was that, despite intentions for embedded practice, AGRIS was perceived as a temporary research project. Thea^1(BPT)^ explained: *“Embed is probably a strong word [laughs]… it's still a trial in terms of its perception”.*

The minimum of nine forms per-month of CONCORDANCE participants, (rather than all eligible admitted patients) seemed the overall target for several sites. Yvette^1(CT Coord)^ explained: *“I collected all the GRACE scales… but they said, ‘Oh no, we only do the first ten per-month’.”* Alice^4(CT Coord)^ struggled for that target: *“They needed nine consecutive admissions … we were struggling, so… [the team] decided… get two or three consecutive patients, for a total nine each month.”*

#### Communication behaviours

No hospital reported the GRT as an embedded element in routine clinical conversations. Regional hospitals described the greatest utility for communication. Jerome^2(AT)^ revealed: “*Having objective measures increases your ability to make an argument… Particularly when we can’t get patients up to bigger hospitals easily.”* From the same facility, Abigail^2(CT Coord/APN)^ recalled: *“I've seen them mention the score in a couple of transfer letters…”,* and *“Some… notes will say, ‘Therefore, we've started [X] treatment’… but not a lot”.*

Cardiology APNs Maree^5^ and Beth^2^ used the GRT in bedside rounds during implementation. Beth^2^ explained: “*You can point out… ‘This patient has a high [GRT]. If we can't get them to Hospital X, can we get them somewhere else?'”* However, few participants recalled its application in this context. The problem seemed to be its unsystematic use. Sam^1(HoD)^ explained, *“We don't have the culture of someone saying, ‘Mrs. Smith's arrived with ACS and her GRACE score is this.’ So it might be calculated, but it's not integrated into handover or clinical pathways*”.

#### Clinical practice behaviours

While participants saw value in the GRT, not many believed it changed clinical practice. Thea^1(BPT)^ explained: “*I don't think it's changed our* [angiography] *practice… checking optimal guideline medical therapy on discharge… that's probably improved… and… cardiac rehab referrals”.* Sandra^3(APN)^ agreed: *“I don't think it's changed what we do. I think it's validated what we do.”*

## Discussion

The COM-B model, underpinned by the Theoretical Domains Framework, was a valuable analytic tool to collect and analyse qualitative data on implementation fidelity. The sources of behaviours (capability; opportunity; motivation) are integrated by its theorists into the “Behaviour Change Wheel” (Fig. 2), a useful vehicle to discuss implementation strategies described by participants. In future GRT implementation initiatives, local implementation strategies could, perhaps, be strengthened by longitudinal stakeholder interviews to inform dynamic, tailored application of the intervention functions and policy categories of the Behaviour Change Wheel in a continuous cycle of practice development. While often seen as *one more thing to do*, the extra work generated by the GRS tool apparently fed into care processes and quality indicator alignment for some participants. Using ongoing stakeholder feedback during implementation may address issues early, raise awareness among junior clinicians and shape their cognitive approach to ongoing patient assessment. This manuscript, through the use of this theoretical structure, provides insight into *how* to improve and promote future implementation of risk assessment into daily practice, and *how* this ongoing, timely feedback may complete the quality improvement cycle.

Perhaps the *intervention functions* most evident were the positive elements of *training, education*, and *persuasion (eg staff meetings, education sessions and incentives)*, and *modelling and leadership* (through *enablement* eg making forms visible, and key clinical advocates supporting/encouraging form completion). These strategic foci likely reflect the investigators’ reasonable assumption that specialist clinicians concerned for patient welfare, presented with compelling evidence, appropriate tools and support would engage with an intervention. Study coordinators were integral to GRT completion and this likely reflects their passion and commitment to the study’s momentum. This was, however, at odds with the study protocol which reserved implementation activities for clinicians. This may explain the wider perception of GRT risk-stratification as a time-limited research activity, rather than an intention for usual care.

The AGRIS steering committee deliberately avoided coercing or legislating change (i.e., mandating), with treatment decisions remaining at the discretion of clinicians. Such flexibility is known to enhance implementation success [33, 34]. Local adaption of GRT layout and treatment recommendations were permissible, providing these remained aligned to process measures for high-risk patients (coronary angiography, evidence-based medications, cardiac rehabilitation). This discretion reflected understanding that risk-assessment tools are designed to complement rather than replace clinical judgement [34]. Regardless, some medical participants described a persisting perception that the GRT (and perhaps decision support tools in general) “mandate” a specific approach rather than guiding/complementing clinical judgement.

Despite this implementation science strategy, it was difficult to embed behaviour change, particularly in EDs where this risk-stratification would ideally occur. This mirrors findings of another Australian process evaluation, the T^3^ trial [35] that aimed to improve triage, treatment and transfer of stroke patients in the ED. Notwithstanding a rigorously-designed implementation program and initial high-level clinician buy-in, contextual factors including beliefs about supporting evidence, and poor medical staff engagement were significant barriers. Perceived *professional boundaries* created tension between the roles and responsibilities of general ED versus specialist stroke practice [35]. This resonates with identified barriers in our study, with a perception of differing evidence bases underpinning cardiology and ED practice, and preferred tools such as the EDAC score for the emergency setting.

Another Australian evaluation, the multi-centre Accelerated Chest pain Risk Evaluation (ACRE) project [36] was initiated by Queensland Emergency clinicians. Using the Theoretical Domains Framework, they also identified *professional roles and boundaries* as a barrier, although with the reverse of our problem: the ED initiators found it difficult to engage Cardiology clinicians, despite obvious overlap in clinical ACS care. They noted higher engagement in facilities with a strong existing collaborative culture between Cardiology and ED.

The perceived impact on workflow during medical rounds, and the sense that formal risk-stratification was ‘one more thing to do’ resonates with past research. ED participants in the T^3^ trial [35] saw the extra clinical activities as time-consuming distractions to more urgent priorities, and the Canadian Head CT rule study [37] found institutional context and resources shaped engagement, citing poor rule compliance when clinicians were particularly busy. In the process evaluation of computerised asthma and angina decision-support [38], it was the complexity and comorbidities of patients that challenged tool integration during busy clinical encounters. This study was used as an exemplar in the UK’s Medical Research Council guideline on developing/evaluating complex interventions [25]. They acknowledged the difficulty gaining traction in such programs, despite a well-conceived implementation and initial enthusiasm and optimism of stakeholders.

Several of our participants cited Chest Pain Pathways as exemplifying a systemic lack of engagement with standardised ACS assessment/decision-support. Despite Chest Pain Pathways mandated as the Australian minimum standard for chest pain evaluation, a “cultural aversion to pathways” and disparities between ED and cardiology commitment are two reported explanations for inconsistent pathway use and significant adverse events in Root Cause Analyses and coronial investigations [39].

Furthermore, a tool considered widely adopted at systems level, but with low levels of use when audited, reveals the dichotomy between care‐as‐practiced and care‐as‐documented [40]: failing to enrol key actors in a tool’s use gives an outside appearance of transparency and standardisation, while preserving medical autonomy that results in process variance with evidence-based care [25]. Without the logical structure of a risk-assessment tool, assessment findings and associated evidence-based decision-making is hard to teach, document and communicate [41] and is difficult to audit for quality review and benchmarking.

Perhaps the greatest potential for formalised risk-assessment tools is the visual nomogram designed to link risk-stratification to clinical decision-making, encouraging systematic deliberation. This may reduce conceptual biases in memory, improving behavioral intentions among less-skilled individuals [42]. If the data required for these common risk stratification tools were built into eMR admission systems as threshold activities (i.e. electronic order sets), feeding into automated risk-calculations and digitally-displayed, evidence-based treatment plans, it could reduce perceived ED workload, increase risk score visibility, and capture important data for quality monitoring/improvement. These should be developed with ED/Cardiology workflow in mind [43]. The groundwork for such technologies is rapidly developing [44, 45].

A limitation of this study is the nature of the sample. Qualitative research most commonly uses a non-probability strategy guided by the characteristics of a population, and the study objectives, and in our case, we did seek maximum variation for discipline and level of experience. In the initial design, the process evaluation was intended to focus on key professionals involved in the local AGRIS implementation. While we planned for implementation to be well-established by the time of recruitment, several trainee physicians and nursing staff involved in initial implementation had moved from their departments/hospitals and were unavailable for interview. A snowball strategy was used because the implementation approach, by design, differed at each site, as did the characteristics of staff involved. While we sought inclusion of people in key roles such as Heads of Department, APNs and medical trainees at each site, the ethical principle of arms-length recruitment meant these invitations were extended via the local study coordinators. This snowball sampling may have introduced selection bias [46]: coordinators likely approached people most motivated by, and committed to, the trial; senior clinicians who felt accountable for an unsuccessful implementation may have been less willing to provide an interview; and, while one APN worked in the emergency setting, the ED clinicians who were difficult to engage in the trial (and therefore would be most informative for this evaluation) were not recruited. Furthermore, in making an initial decision not to engage, they may not have viewed themselves as stakeholders. Finally, the opinions of rotating medical officers in control arm facilities without access to decision-support would be useful to access, given the GRT is considered most valuable for less experienced physicians.

## Conclusions

Despite implementation leadership from local AGRIS champions, poor wider implementation fidelity reflected a failure to embed risk-stratification processes into routine admission procedures, clinical conversations and a documented rationale for practice throughout the ED and early cardiology patient journey. This understanding may, in part, explain the neutral AGRIS results, while providing valuable insights into future implementation initiatives. Strategies that strengthen collaboration with EDs and integrate automated processes for risk-stratification data may improve future translation. The English (UKGRIS) and planned Canadian (CanGRIS) studies will provide larger samples, and more conclusive understandings of the potential for routine risk-stratification in improving ACS care [14].

## Data Availability

The datasets generated and analysed during the current study are not publicly available. Raw qualitative data contains unique stories that if shared, threatens participant confidentiality. As this is workplace data, many participants are known to each other. We do not have participant consent to share raw data. Additionally, the context-bond nature of qualitative data limits its use for secondary analysis when removed from its context.

## Abbreviations

ACS: Acute Coronary Syndrome
AGRIS: Australian Grace Risk Score Intervention Study
APN: Advanced Practice Nurse
AT: Advanced Physician Trainee
BPT: Basic Physician Trainee
COM-B Model: Capability, Opportunity, Motivation – Behaviour Model
CT Coord: Clinical Trials Coordinator
ED: Emergency Department
GRACE: Global Registry of Acute Coronary Events
GRT: Grace Risk Tool
STEMI: ST Elevation Myocardial Infarction
MI: Myocardial infarction
NSTEMI: Non-ST Elevation Myocardial Infarction
T^3^ trial: Triage, treatment and transfer of patients with stroke in emergency departments trial
UKGRIS: United Kingdom GRACE Risk Intervention Study
CanGRIS: Canadian GRACE Risk Intervention Study
